# Influence of Machining Allowance, Build Orientation, and Cutting Parameters on Hole Quality in Additively Manufactured ABS Components

**DOI:** 10.3390/ma19153173

**Published:** 2026-07-24

**Authors:** Artur Szajna, Tomasz Rydzak, Anna Bazan, Paweł Turek, Andrzej Kawalec, Mario Álvarez-Blanco, Antonio Guerra-Sancho

**Affiliations:** 1 Department of Manufacturing Techniques and Automation, Faculty of Mechanical Engineering and Aeronautics, Rzeszów University of Technology, 35-029 Rzeszów, Poland; a.szajna@prz.edu.pl (A.S.); t.rydzak@prz.edu.pl (T.R.); pturek@prz.edu.pl (P.T.); ak@prz.edu.pl (A.K.); 2 Department of Mechanical Engineering, Universidad Carlos III de Madrid, Avenida de la Universidad 30, 28911 Leganés, Spain; marioab@ing.uc3m.es (M.Á.-B.); antguerr@ing.uc3m.es (A.G.-S.)

**Keywords:** Material Extrusion, ABS polymer, drilling, dimensional accuracy, surface roughness, hybrid manufacturing

## Abstract

Material Extrusion (MEX) additive manufacturing (AM) of ABS polymer components often requires post-process machining to achieve the necessary dimensional precision and surface quality. However, the influence of printing parameters and tool–material interaction in hybrid manufacturing remains insufficiently explored. This study investigates the impact of initial hole size (D_start_), build orientation, and cutting parameters (cutting speed and feed rate) on the dimensional accuracy and surface roughness of machined holes in ABS-M30 specimens. Samples were fabricated in vertical and horizontal orientations and subjected to drilling in solid material and enlargement of printed pilot holes using a twist drill on a 5-axis machining center. Dimensional deviation and surface roughness (Ra, Rz) were evaluated using coordinate metrology and profilometry. The results showed that the smallest machining allowance (0.062 mm per side) was insufficient to completely remove the printing-induced surface texture, resulting in significantly higher and more variable Ra and Rz values. This distinct low-machining-allowance regime was confirmed by statistical analysis and representative optical observations. Conversely, a machining allowance of 0.565 mm per side (corresponding to D_start_ = 9 mm) resulted in substantially lower surface roughness (Ra ≈ 1.6 µm). Vertical build orientation generally provided better surface quality than the horizontal orientation, which was consistent with fewer visible surface features in the selected optical fields of view. All machining conditions resulted in negative dimensional deviations, indicating elastic recovery of the ABS material after machining. An exploratory multi-response ranking showed that the lowest composite quality scores for overall final hole quality were associated with D_start_ = 10 mm (machining allowance of 0.062 mm per side). When the analysis was limited to the machining-dominated regime, the lowest score was obtained for the vertical build orientation, D_start_ = 9 mm, a cutting speed of 40 m/min, and a feed rate of 0.2 mm/rev. These findings provide preliminary guidelines for selecting hybrid manufacturing conditions for MEX-manufactured ABS-M30 components.

## 1. Introduction

Material Extrusion (MEX) additive technologies have become a widely used tool for rapid prototyping and low-volume production [1,2]. Acrylonitrile–butadiene–styrene (ABS) remains one of the most commonly used polymers due to its favorable mechanical properties, ease of processing, and relatively high thermal resistance [3,4]. Consequently, MEX-manufactured ABS components are increasingly used in demanding applications such as aerospace, automotive, and medical engineering [5,6,7,8]. However, manufactured prints often require post-processing operations, including drilling and enlargement of printed pilot holes, to achieve the dimensional and geometric accuracy required for assembly.

Dimensional accuracy of MEX components is mainly affected by layer anisotropy, thermal shrinkage, extrusion path segmentation and process parameters such as layer height, print speed, nozzle temperature, infill density, shell number and build orientation [8,9,10,11,12,13,14,15,16,17]. Among all geometrical features, holes and cylindrical surfaces are particularly susceptible to dimensional errors caused by the stair-stepping effect, often resulting in undersized diameters and deviations from circularity [18,19].

To compensate for these limitations, printed holes are commonly finished by drilling or enlargement of printed pilot holes. Existing studies on machining of MEX polymers mainly concern PLA and its composites [20,21,22,23]. These studies demonstrate that high feed rates and large drill diameters increase cutting forces, delamination and surface roughness, whereas higher cutting speeds generally improve hole quality and reduce cutting forces. Thermal phenomena occurring during the machining of polymers must also be taken into account, as the material’s low thermal conductivity, combined with excessively high cutting parameters, can lead to local melting of the material on the hole walls [23]. Owing to the layered and anisotropic structure of MEX-manufactured parts, machining mechanisms differ from those observed in conventionally manufactured polymers, making direct transfer of machining recommendations inappropriate [14].

Despite the widespread use of ABS in Material Extrusion, previous studies have mainly examined either the influence of printing parameters on the quality of printed features or the machining behaviour of other MEX polymers, particularly PLA [20,21,22,23,24]. Consequently, little is known about how the additive manufacturing process and subsequent drilling interact to determine the final quality of machined holes in ABS-M30 components. In particular, the combined effects of build orientation, printed pilot-hole diameter (machining allowance), cutting speed and feed rate on hole quality have not been systematically investigated.

To address this knowledge gap, the present study systematically investigates the combined influence of build orientation, printed pilot-hole diameter (expressed as machining allowance), cutting speed and feed rate on the quality of drilled holes in ABS-M30 specimens manufactured by MEX. Unlike previous studies, which primarily focused on individual printing parameters, directly printed hole accuracy, or machining of PLA-based materials, the present work evaluates the interaction between additive manufacturing and subsequent machining. Hole quality is assessed using three complementary response variables: dimensional deviation, cylindricity, and surface roughness (Ra and Rz). The results provide a basis for future optimization of hybrid additive–subtractive manufacturing of ABS-M30 components.

## 2. Materials and Methods

The aim of the study was to analyze the effects of selected factors on the quality of holes produced either by drilling in solid material or by enlarging printed pilot holes in MEX-manufactured ABS specimens. The experiment was designed based on a full factorial design with the input variables presented in Table 1.

### 2.1. Modeling and Fabrication of Research Samples

The preparation and fabrication process of the experimental samples was carried out in several complementary stages, including CAD geometric modeling, control code preparation (technological preprocessing), and the actual additive manufacturing process using MEX technology.

The three-dimensional geometric model of the sample was designed in the Siemens NX CAD environment. The research object had a rectangular cuboid shape with nominal external dimensions of 125 mm × 25 mm × 15 mm. The structure of the samples included four printed pilot holes with varying nominal diameters: 9.0 mm, 9.5 mm, 9.8 mm, and 10.0 mm, as well as a solid material section designated for drilling in solid material (without a pilot hole), in accordance with the adopted experimental design (Table 1).

Subsequently, the digital solid model was tessellated and exported to the Stereolithography (STL) format, which defines the polygonal representation of the object’s surface. To minimize the adverse effect of approximation errors on the subsequent dimensional accuracy of the cylindrical holes, strict mesh generation parameters were applied in the Siemens NX software (version 2212). The chord tolerance was set to 0.01 mm, while the angular tolerance was restricted to 1.0°. The application of such advanced discretization criteria allowed for a smooth reproduction of arcs and cylinders, reducing shape discretization errors to values significantly lower than the positioning resolution and the nozzle bead error of the industrial 3D printer. This eliminated the risk of the polygonal geometry influencing the cutting force parameters and roundness deviations during subsequent drilling and enlargement of printed pilot holes.

**Figure 1:**
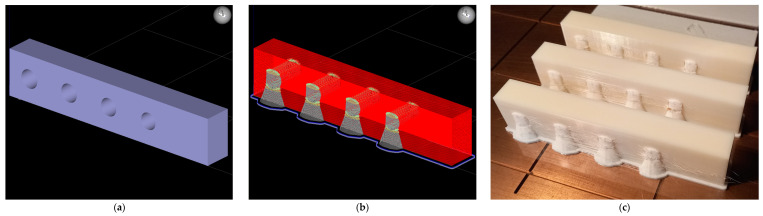
Process of preparing and fabricating the research model in a horizontal orientation: (**a**) model imported into the Insight software; (**b**) model with generated support material and sliced into layers; (**c**) model manufactured on the Fortus 360mc 3D printer (Stratasys, Eden Prairie, MN, USA).

The STL file was imported into the specialized technological software Insight (version 16.1) (Stratasys), dedicated to industrial additive manufacturing systems (Figure 1a). Within this environment, sample orientation in the machine work volume, slicing into layers, and toolpath generation for the extrusion nozzle were performed. In accordance with the research assumptions, the samples were oriented on the build platform in two build arrangements to evaluate the effect of layer structure anisotropy on the geometric errors of the holes after machining:

- Vertical orientation: where the hole axes were positioned perpendicularly to the XY build plane (parallel to the Z-axis);
- Horizontal orientation: where the hole axes were positioned parallel to the XY build plane.

In the toolpath generation process (Figure 1b), a solid internal fill was specified. A solid infill setting and zero nominal raster air gap were used to promote a dense internal structure and reduce inter-path gaps. These process settings do not demonstrate the complete absence of local voids or variations in inter-path bonding. During the configuration of the toolpath parameters, the nominal outer contour width was set to 0.3556 mm, and the visible surface finish style was defined as Enhanced to ensure the highest possible density and the finest reproduction of the external layer of the printed specimens. For geometries requiring structural support (particularly in the horizontal orientation), sparse support structures were generated. To facilitate the subsequent removal of mechanical supports, an easy-separation option (Break-away) was implemented.

The research samples were fabricated using a Stratasys Fortus 360mc industrial production system (Figure 1c). Amorphous thermoplastic ABS-M30 (acrylonitrile butadiene styrene) in ivory was used as the model material, which features enhanced mechanical strength and interlayer cohesion compared to standard ABS. For the construction of support structures necessary to properly reproduce the hole geometry in the horizontal orientation, SR-30 soluble support material was utilized. Material deposition for both the model and support was carried out using standard T12 extrusion nozzles (nozzle diameter 0.3 mm). The selection of the Stratasys Fortus 360mc platform and ABS-M30 material was dictated by the system’s industrial repeatability and the material’s superior interlayer adhesion, which has been extensively documented in comparative studies [25]. Given the inherent anisotropy of the MEX technology [26], the chosen fabrication strategy aimed to minimize the impact of build orientation on the final geometric integrity of the machined features, as highlighted in previous research regarding the post-processing of thermoplastic components [27].

Based on the configuration in the Insight software, fixed process parameters for the sample fabrication were established and are summarized in Table 2.

The strategy of material deposition involved printing the outer contours first, followed by filling the interior using linear raster toolpaths oriented at +45° and −45° relative to the part axis, alternating the direction by 90° in each subsequent layer (cross-hatched layer structure). The application of a zero raster air gap (Raster air gap = 0.0000 mm) was intended to promote a comparatively dense and cohesive structure, which is critical for the repeatability and consistency of subsequent destructive machining processes.

Upon completion of the production cycle, the samples along with the build sheet were removed from the printer work chamber. The support structures were then removed via chemical dissolution in a dedicated cleaning solution using a water-chemical circulation system, yielding the final specimens for further experimental testing.

### 2.2. Experimental Investigation of Drilling and Enlargement of Printed Pilot Holes

The subtractive machining process of holes (comprising both drilling in solid material and the enlargement of printed pilot holes) was carried out under strict procedures to ensure high repeatability of the experimental conditions.

The machining trials were conducted on a modern 5-axis vertical machining center, the DMU 100 MonoBlock (DMG MORI, Bielefeld, Germany), which features high static and dynamic rigidity of the Machine Tool-Workpiece-Fixture-Tool system. The test specimens made of ABS-M30 material were clamped in a precision machine vise mounted directly onto the worktable of the machine tool.

To eliminate datum setup errors and ensure the repeatability of the workpiece position along the vertical Z-axis, precision-ground parallel inserts were utilized. They enabled repeatable seating of the specimens at a constant, strictly defined depth, ensuring that the entire gripping height of the machined part (15 mm) was located directly between the vise jaws. To minimize the effect of clamping force variation on the elastic deformation of the compliant thermoplastic material, a torque wrench calibrated to a constant torque value was used for each clamping cycle [27].

A standard twist drill with a nominal diameter of D = 10 mm made of high-speed steel (HSS) was selected as the machining tool. The tool was mounted in a collet chuck with an ER32 collet, guaranteeing a high gripping force on the cylindrical shank. Prior to testing, the assembled tool unit (chuck and drill) underwent precise metrological verification on a Zoller smarTcheck laser tool presetter (E. Zoller GmbH & Co. KG, Pleidelsheim, Germany). The measurement indicated that the effective cutting diameter of the tool, taking into account its radial runout, was 10.08 mm.

**Figure 2:**
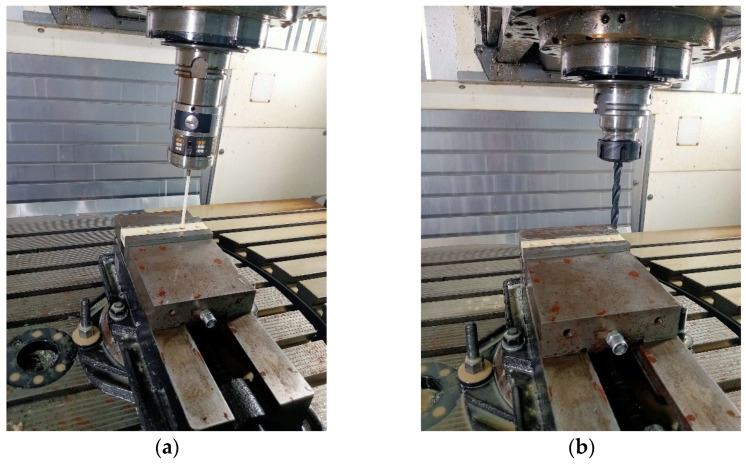
Experimental procedure on the DMU 100 MonoBlock machining center: (**a**) identification of the geometric center of the hole using a touch-trigger workpiece probe; (**b**) enlargement of printed pilot holes with flood cooling applied.

Before starting each cutting cycle, an automatic routine was performed to identify the geometric center of the printed pilot hole (or the reference datum point for drilling in solid material). This operation was carried out using a high-precision, touch-trigger workpiece probe (Figure 2a) included as standard equipment on the machining center. This eliminated concentricity errors (positioning errors along the X and Y axes), ensuring that the tool’s rotation axis was perfectly concentric with the geometric center of the pilot hole in every test.

Machining was performed with a constant, continuous working feed across the entire depth of the sample, producing through-holes. Due to the specific nature of polymer subtractive manufacturing (the tendency of ABS to undergo severe thermal deformation and cause chip clogging in the flutes), intensive flood cooling was applied [28]. In the present study, an HSS twist drill without internal coolant channels was used. Therefore, external flood cooling was applied as the only available and technically feasible cooling strategy for the selected tool and machine-tool configuration. A 7% water–oil emulsion based on Fuchs Ecocool Global 1000 concentrate was used as the cutting fluid (coolant). The individual combinations of cutting parameters for each experimental run, determined by the full factorial design, are specified in Table 3. The investigated cutting-speed (v_c_ = 20, 30, 40 m/min) and feed-rate (f = 0.1, 0.2, 0.3 mm/rev) ranges were selected with reference to previous studies on the drilling of MEX-manufactured thermoplastics [14,20,22] and were subsequently verified in preliminary machining trials conducted under flood-cooling conditions. The preliminary trials indicated that stable machining could be achieved within the selected range without excessive thermal softening, material adhesion to the cutting tool, or chip clogging. These preliminary trials were used solely to identify a technically stable parameter range and were not included in the statistical analysis. Lower cutting parameters promoted unstable material removal, whereas higher values increased the risk of thermal softening and material smearing.

The tool path cycle was programmed as follows:

- Rapid approach along the Z-axis to a safe clearance distance above the top surface of the specimen;
- Feed motion at the programmed feed rate and cutting speed to the full depth of the part (through-hole penetration);
- Upon reaching the target end point, a programmed dwell time of exactly 0.5 s was introduced. This aimed to fully form the exit edge of the hole and completely evacuate any remaining chips;
- Rapid retraction of the tool along the Z-axis above the top surface of the specimen.

The experimental plan was carried out using 18 rectangular ABS-M30 specimens.

In total, nine specimens were manufactured in the horizontal build orientation and nine in the vertical build orientation. For each build orientation, the nine specimens corresponded to the complete 3 × 3 factorial combination of cutting speed and feed rate. Each specimen contained five hole conditions: one location for drilling from solid material and four printed pilot holes with nominal diameters of 9.0, 9.5, 9.8, and 10.0 mm. The physical positions of the five hole conditions were fixed and identical across all specimens. Consequently, the printed pilot-hole condition and physical hole position were confounded, and their individual effects cannot be separated within the present experimental design. The experimental dataset comprised 90 machined holes in total. Although independent replications of the manufacturing and machining process were not performed, the full factorial design and repeated metrological measurements allowed the main technological trends to be identified under controlled conditions. A restricted block-wise randomization strategy was applied. The order of machining the specimens was randomized. After each specimen was clamped, all five holes were machined within the same setup in order to avoid additional setup and clamping variability. Within each specimen, the order of machining the five hole conditions was also randomized. Thus, the specimen/clamping setup was treated as a practical block, while the hole machining order within each block was randomized.

### 2.3. Dimensional and Geometric Accuracy and Surface Texture Measurements

To quantitatively evaluate the effect of the analyzed technological factors on the quality of the machined holes, all experimental samples underwent rigorous metrological verification. The scope of the investigation included the assessment of macrogeometric accuracy (dimensional deviation of the diameter) as well as microgeometric characterization (surface roughness). Due to the high thermal expansion coefficient and mechanical compliance of the amorphous ABS-M30 thermoplastic material, all measurement procedures were conducted in a metrology laboratory with controlled and monitored environmental conditions. Following the machining processes, the specimens were subjected to a 24 h climatic stabilization period at a constant temperature of 20 ± 0.5 °C and a relative air humidity of 50 ± 5%, which is consistent with established protocols for minimizing thermal drift in additively manufactured polymeric parts [29].

**Figure 3:**
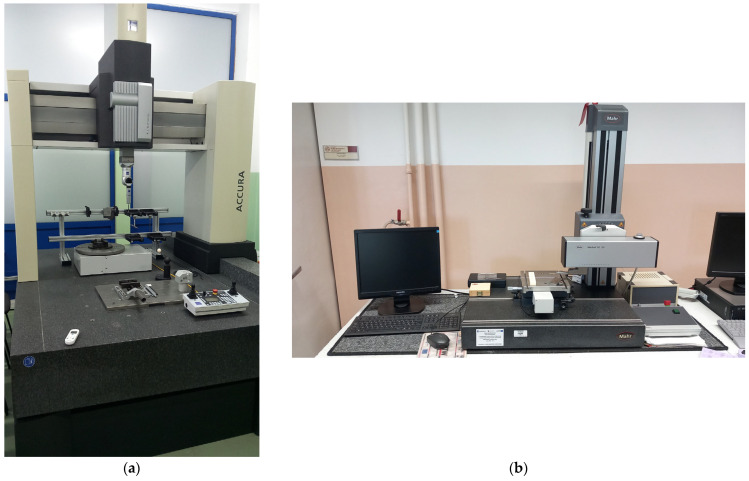
Measurement systems: (**a**) Carl Zeiss Accura II; (**b**) MarSurf XR 20 profilometer.

The diameters of the holes, both before and after the drilling process, were measured using a Carl Zeiss Accura II (ZEISS, Oberkochen, Germany) (Figure 3a) coordinate measuring machine (CMM) equipped with a VAST XXT touch-trigger scanning probe (ZEISS, Oberkochen, Germany) and Calypso software version 2022. The measurement strategy was optimized to minimize errors arising from the elasticity of the polymer material; specifically, a reduced measuring contact force (0.05 N) was applied, which successfully prevented local indentation of the Ø 3 mm ruby stylus ball into the layered ABS structure. Minimizing the probing force is critical when executing tactile metrology on compliant polymer structures to eliminate significant elastic–plastic deformation at the contact point [30]. The measurement of each hole’s dimension was executed using a continuous circular scanning path across three distinct cross-sections (measuring planes) located at varying depths along the hole axis:

- Section A (entry zone): Z = −3.0 mm from the top face of the specimen;
- Section B (middle zone): Z = −7.5 mm from the top face of the specimen;
- Section C (exit zone): Z = −12.0 mm from the top face of the specimen.

Based on the captured point clouds (sampling density: 50 points per mm), the following diagnostic parameters were calculated using the Least Squares Circle (LSC) evaluation algorithm:

- Dimensional deviation (Err): the dimensional deviation was calculated with respect to the effective drill diameter (10.08 mm, including tool runout), rather than the nominal drill diameter of 10.00 mm. This approach was adopted because the effective drill diameter represents the actual cutting geometry and therefore provides a more appropriate reference for interpreting machining-induced dimensional deviations.
- Cylindricity (Cyl): determined via three-dimensional correlation of all three measuring planes in compliance with the ISO 12180 parts 1 and 2 standard [31,32].

The evaluation of the internal hole surface roughness was conducted using an advanced MarSurf XR 20 profilometer system (Mahr GmbH, Göttingen, Germany) (Figure 3b) coupled with a MarSurf GD 120 drive unit (Mahr GmbH, Göttingen, Germany). The measurements were performed using a stylus tip featuring a nominal radius of 2 µm. The assessment was executed along the generating line of the hole (perpendicular to the tool machining marks and parallel to the hole axis) at six locations. In accordance with the guidelines of the ISO 21920 standard [33], the following measurement parameters were established:

- Cut-off length: λ = 0.8 mm;
- Evaluation length: l_n_ = 4.0 mm;
- Profile filter type: Phase-correct digital Gaussian filter.

The application of a phase-correct digital filter ensured accurate separation of high-frequency roughness from macro-geometric waviness without introducing phase shifts [34]. The primary indicators subjected to statistical analysis were Ra (arithmetical mean deviation of the roughness profile) and Rz (maximum height of the roughness profile), which constitute the most reliable descriptors for evaluating the surface integrity of additively manufactured thermoplastics after post-processing operations [35].

### 2.4. Statistical Analysis

Statistical analysis was performed using both the R and Python programming languages with the appropriate libraries [36,37,38,39]. For all statistical tests conducted, a significance level of 0.05 was assumed.

Statistical analysis was performed using both the R (version 4.3.2) and Python (version 3.11.5) programming languages with the appropriate libraries [36,37,38,39]. For all statistical tests conducted, a significance level of 0.05 was assumed. Linear mixed-effects models were initially fitted including all main effects and two-way interactions. To account for the dependence of the hole conditions measured within the same specimen, specimen identity was included as a random intercept, whereas build orientation, cutting speed, feed rate, and D_start_ were treated as fixed effects. Model simplification was subsequently performed using backward elimination implemented with the step() function from the lmerTest package in R. The final models retained all corresponding main effects whenever interaction terms were present, thus preserving model hierarchy. Model fit was summarized using marginal R^2^, representing the variance explained by the fixed effects, and conditional R^2^, representing the variance explained jointly by the fixed and random effects.

For the regression analysis, all experimental conditions were initially considered. However, the exploratory data analysis revealed that the D_start_ = 10 mm condition, corresponding to the smallest machining allowance (0.062 mm per side), exhibited distinctly different machining behaviour compared with the remaining conditions and therefore represented a separate machining regime. Consequently, it was retained in the descriptive analysis but excluded from the regression model development to avoid combining fundamentally different machining regimes.

## 3. Results

Results are presented for diameter deviation (Err), cylindricity (Cyl), and surface roughness (Ra and Rz) as functions of build orientation, D_start,_ cutting speed, and feed rate.

### 3.1. Analysis of Dimensional Deviations

The diameters of the 3D-printed holes, in both horizontal and vertical orientations, were smaller by approximately 0.05 mm compared to the nominal values (Table 4). In the remainder of this paper, for the sake of simplicity when describing the results, the nominal values of the pilot holes will be used, where the absence of a hole will be designated as a 0 mm hole. To better represent the actual amount of material removed during drilling, the machining allowance per side was determined from the measured mean diameter of the printed pilot holes and the effective drill diameter (10.08 mm, including tool runout). The calculated allowance values for each nominal pilot-hole diameter are also summarized in Table 4.

All observed diameter deviations (Err) after drilling were negative (Table 5), meaning that the measured diameters were smaller than the nominal value. This is due to the deflection (pushing back) of the material during drilling, which causes the uncut chip thickness to be smaller than it would be for a highly rigid material/sample.

**Figure 4:**
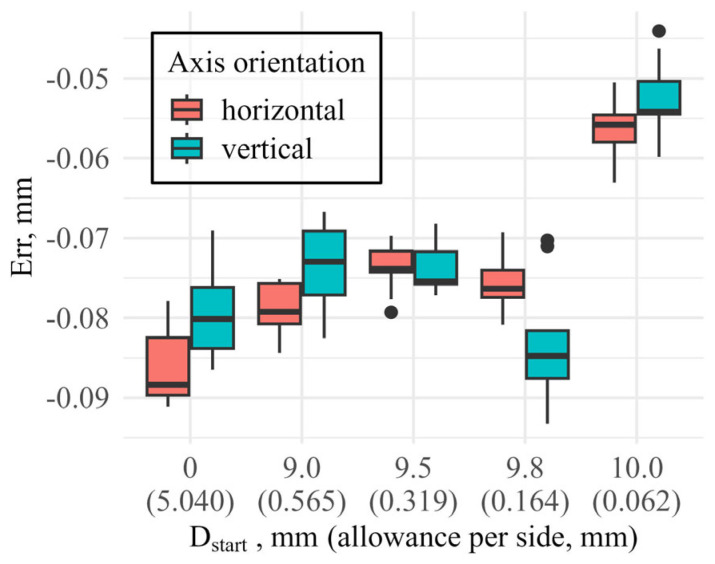
Box plot of diameter deviation (Err) values by pilot hole diameter and orientation (each boxplot includes all cutting conditions).

Due to the relatively small uncut chip thickness, the cutting conditions for the D_start_ = 10 mm (allowance = 0.062 mm per side) samples were sufficiently different to be reflected in significant differences in the deviation values (Figure 4). The box plot shows that the dataset for the 10 mm samples is clearly distinct from the other samples. This suggests that the cutting conditions for this case were significantly different from the rest. For this reason, data regarding the D_start_ = 10 mm samples were excluded when developing the regression model. This interpretation was supported by within-specimen paired comparisons between the D_start_ = 10 mm condition and the mean of the remaining hole conditions, which showed that the D_start_ = 10 mm (allowance = 0.062 mm per side) condition differed significantly from all remaining conditions (*p* = 7 × 10^−13^), indicating that the smallest machining allowance represents a distinct machining regime.

**Figure 5:**
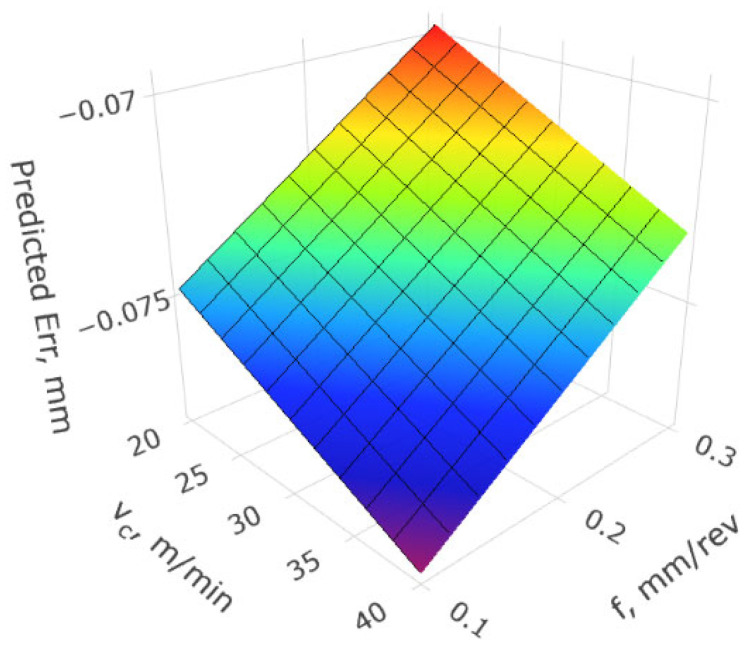
Response surface for the developed regression model of the diameter deviation Err (for vertical orientation and D_start_ = 9.5 mm).

**Figure 6:**
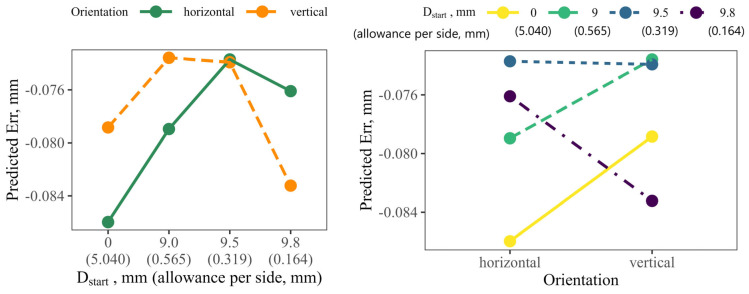
Interaction plots between print orientation and pilot hole size (D_start_) for the response variable Err.

To further investigate the effect of machining parameters on the diameter deviation, a regression analysis was performed, treating D_start_ as a nominal variable due to the substantial differences in D_start_ values between the absence of a hole and the other cases. The model included both main effects and interaction effects. As a result of backward elimination, a statistically significant model was obtained (*p* < 0.001; residual analysis showed no significant deviations from normality or heteroscedasticity). The marginal R^2^, representing the variance explained by the fixed effects, was 0.61, whereas the conditional R^2^, representing the variance explained jointly by the fixed and random effects, was 0.72. The fixed effects explained approximately 61% of the variability in Err, while the inclusion of the specimen-level random effect increased the explained variability to 72%. The model is summarized in Table 6. The response surface developed based on this model is shown in Figure 5. The interactions included in the model are presented graphically in Figure 6.

The intercept has a negative value, which results from the average value of Err that, as previously noted, is also negative. Compared to the baseline hole D_start_ = 0 mm, the largest differences were observed for D_start_ = 9.5 mm. Print orientation significantly affected diameter deviation, although its effect also depended on D_start._ Across the investigated range, increasing cutting speed shifted the predicted diameter deviation toward zero, whereas increasing feed resulted in more negative deviations (Figure 5).

D_start_ × orientation interaction: For the 9.5 mm pilot hole, the deviations had a similar value regardless of the model orientation. For the models without a pilot hole (D_start_ = 0 mm) and for those with 9 mm holes—i.e., for the samples with the largest width of cut—the model predicts smaller deviations (in terms of absolute value) for the vertical orientation. This relationship is visible in the interaction plot (Figure 6) and the box plot in Figure 4.

### 3.2. Analysis of Cylindricity Deviation

**Figure 7:**
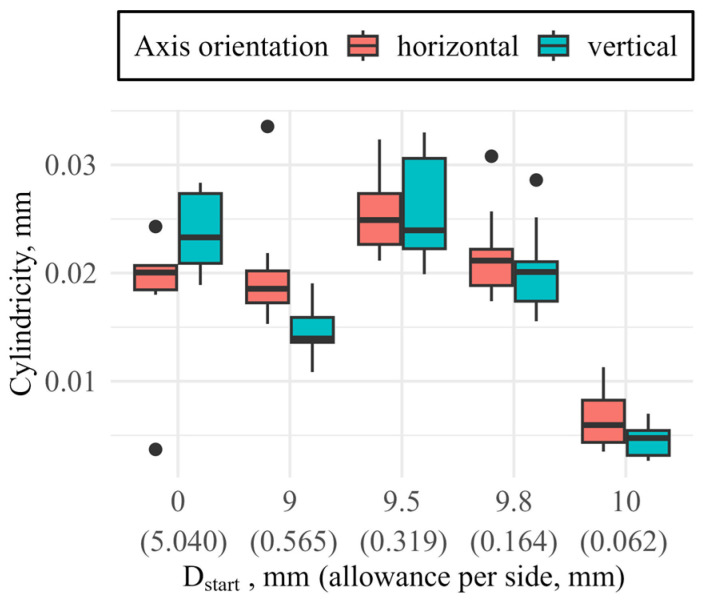
Box plot of cylindricity deviation values by pilot hole diameter and orientation.

The analysis of cylindricity deviation (Cyl) as a function of the D_start_ parameter revealed a clear differentiation of results between the groups. For the D_start_ = 0–9.8 mm range, the mean values of the cylindricity deviation were relatively close, falling within the range of approximately 0.017–0.026 mm (Table 7, Figure 7). The variability of the results remained at a similar level, with a standard deviation on the order of 0.004–0.005 mm. A distinctly different behavior was recorded for D_start_ = 10 mm (allowance = 0.062 mm per side), where the mean cylindricity deviation dropped to 0.006 mm, representing a 3- to 4-fold reduction compared to the other cases. Concurrently, a significant decrease in the variability of the results, expressed by the standard deviation, was observed by approximately 2–2.5 times. This interpretation was supported by within-specimen paired comparisons between the D_start_ = 10 mm condition and the mean of the remaining hole conditions, which demonstrated that the D_start_ = 10 mm (allowance = 0.062 mm per side) condition differed significantly from the remaining machining conditions (*p* = 2 × 10^−15^), supporting its treatment as a distinct machining regime. Due to the different nature of the D_start_ = 10 mm samples, the regression models subsequently developed did not include data from these samples.

To investigate the effect of cutting speed, feed rate, pilot hole diameter, and model orientation on the deviation value, models accounting for main effects and interactions between factors were constructed. As a result of backward elimination, a statistically significant model was obtained. Both the marginal and conditional R^2^ values were 0.44. The fixed effects explained approximately 44% of the variability in Cyl, and inclusion of the specimen-level random effect did not increase the explained variability. Residual analysis showed no significant deviations from normality or heteroscedasticity. The model parameters and the evaluation of coefficient significance are presented in Table 8.

**Figure 8:**
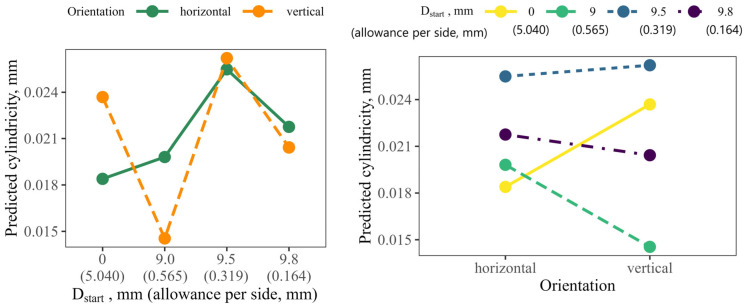
Interaction plots between print orientation and pilot hole size (D_start_) for the response variable cylindricity.

The significant factors proved to be the pilot hole diameter, orientation, and the interaction between these factors (Figure 8). Both R-squared goodness-of-fit values of 0.44 indicate a moderate ability of the model to describe the trends between the studied factors and the final hole cylindricity. The developed model does not explain the majority (56%) of the variance in the dataset of cylindricity deviation values. For two out of the four analyzed D_start_ sizes (i.e., for half of the cases), smaller cylindricity deviation values were observed for the vertical orientation, while for the other half, they were observed for the horizontal orientation. Furthermore, no monotonic relationship was observed between D_start_ and the Cyl value. Based on the conducted study, it is not possible to conclusively recommend either of the orientations or pilot hole values.

### 3.3. Surface Roughness Parameters Analysis

**Figure 9:**
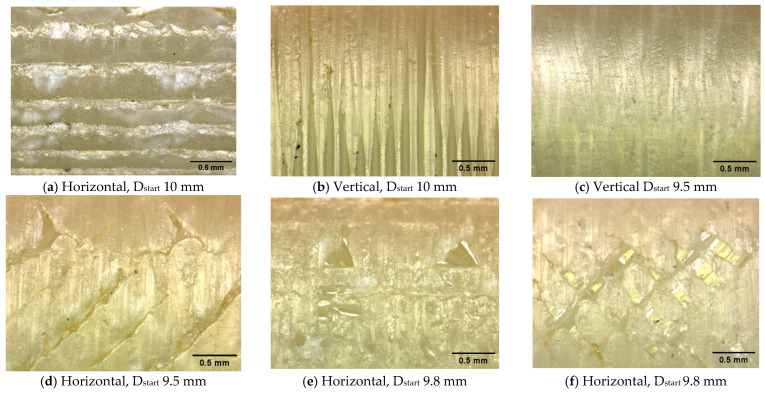
Qualitative optical observations of the machined hole surfaces: (**a**,**b**) residual printing-induced surface irregularities (scallops) remaining after machining; (**c**) surface exhibiting good machining quality; (**d**) feature interpreted as a possible crack; (**e**) local surface damage, including tear-out-like surface features and features consistent with locally adhered material (lower left corner); (**f**) surface feature consistent with material smearing.

During the roughness measurements, it was observed that for the D_start_ = 10 mm condition (machining allowance = 0.062 mm per side), the machining process did not completely remove the surface irregularities originating from the MEX process. Residual printing-induced scallops are visible in the qualitative optical observations shown in Figure 9a,b. In general, the holes produced in specimens printed in the vertical orientation exhibited better surface quality and fewer visible surface features in the selected optical fields of view than those printed in the horizontal orientation. Qualitatively observed surface features included line-like discontinuities oriented approximately at 45° to the hole axis, tear-out-like features, locally adhered material, and surface features consistent with material smearing.

**Figure 10:**
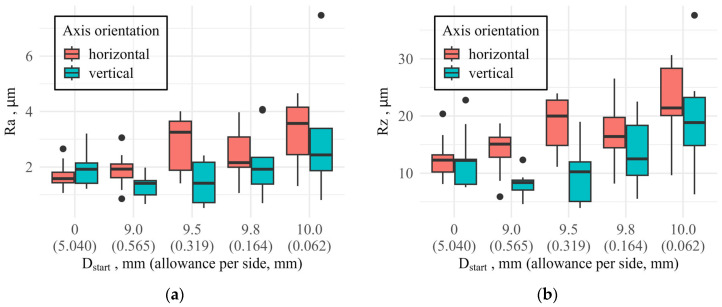
Box plot of: (**a**) Ra; (**b**) Rz parameter values by pilot hole diameter.

The observations described above are reflected in the measured surface roughness values (Table 9 and Table 10, Figure 10). Within a single box, values obtained for different cutting parameters are presented. As previously noted, for the D_start_ = 10 mm samples, these residual surface features contributed to the increased Ra and Rz values. The D_start_ = 10 mm condition was excluded from the regression analysis because the objective of the modelling was to evaluate the surface roughness generated by machining. It should also be noted that for specimens printed in the horizontal orientation, the measured Ra and Rz values may be underestimated because the profilometer stylus traversed parallel to the deposited layers, making the measurement less sensitive to the residual layer profile.

**Figure 11:**
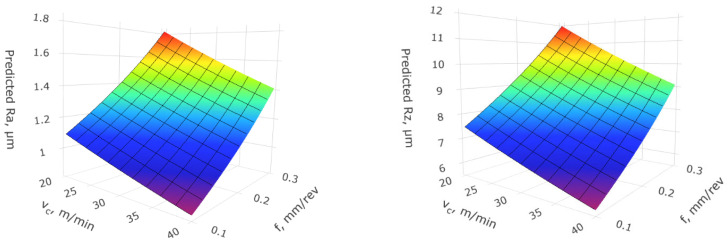
Response surface for the developed regression model for Ra and Rz (for vertical orientation and D_start_ = 9.5 mm).

This translated into higher values of surface roughness amplitude parameters (Table 9 and Table 10 and Figure 11). Within a single box, values for different cutting parameters are presented. It can be observed that the D_start_ = 10 mm samples (allowance = 0.062 mm per side) were characterized by a significantly higher variability than the others. For these samples, it was also observed that the removed allowance was so small that the scallops between the deposited material layers formed during the printing process remained. The residual scallops likely contributed to the higher Ra and Rz values. This interpretation was supported by within-specimen paired comparisons between the D_start_ = 10 mm condition and the mean of the remaining hole conditions, both for Ra (*p* = 0.004) and Rz (*p* = 3 × 10^−4^). The D_start_ = 10 mm condition was excluded from the regression analysis because the objective of the modelling was to evaluate the surface roughness generated by machining. For this condition, the machining allowance was insufficient to completely remove the printing-induced surface irregularities, meaning that the measured roughness reflected both the original MEX surface and the subsequent machining process.

The qualitative surface evidence presented in Figure 9 is also consistent with the lower Ra and Rz values obtained for the vertical build orientation, which may be attributed to fewer visible surface features on these surfaces.

The dispersion of the values shown in Figure 10 suggests that the variability across the pooled cutting conditions depended on the machining allowance. The cutting parameters had the least effect on surface roughness for the two largest machining allowances (D_start_ = 0 mm and D_start_ = 9.0 mm).

To investigate the effect of machining parameters on surface roughness described by the Ra parameter, a regression analysis was performed for the variables log(Ra) and log(Rz) as a function of the following parameters: cutting speed, feed rate, pilot hole diameter, and model orientation. The values of the response variables were log-transformed to satisfy the analysis of variance (ANOVA) assumptions for regression. The models accounted for main effects and interactions between factors. Backward elimination yielded statistically significant models. For log(Ra), both the marginal and conditional R^2^ values were 0.49. The fixed effects explained approximately 49% of the variability in log(Ra), and inclusion of the specimen-level random effect did not increase the explained variance. For log(Rz), the marginal R^2^ was 0.59 and the conditional R^2^ was 0.65. Residual analysis showed no significant deviations from normality or homoscedasticity. The model parameters and the evaluation of coefficient significance are presented in Table 11 and Table 12.

Based on the standardized coefficient values, it can be inferred that the feed rate, the D_start_ value, and its interaction with the build orientation had the greatest effect on the Ra and Rz values. The main effects of v_c_ and f also proved to be statistically significant factors, whereas the effect of build orientation was significant only through its interaction with the initial hole diameter. An increase in the feed rate resulted in an increase in both Ra and Rz values across the entire analyzed experimental space. Conversely, an increase in the cutting speed resulted in a decrease in both roughness parameters (Figure 11).

**Figure 12:**
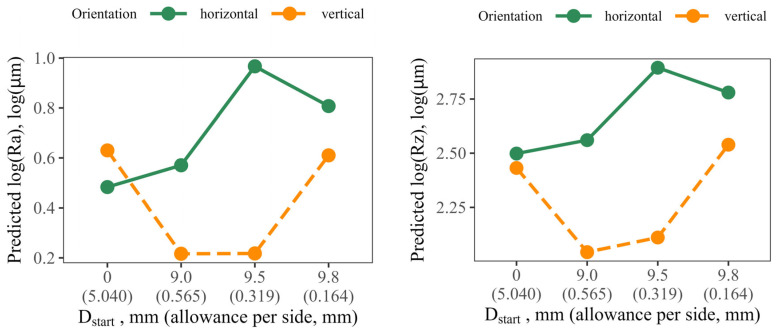
Interaction plots between print orientation and pilot hole size (D_start_) for the response variables log(Ra) and log(Rz).

In the case of drilling from solid material (D_start_ = 0 mm), the build orientation did not have a significant effect on the roughness parameters. For the 9 mm and 9.5 mm pilot hole sizes, the vertical orientation was more favorable; i.e., it was associated with lower hole roughness after enlargement of printed pilot holes (Figure 12).

In general, the lowest values of roughness parameters were obtained for the vertical orientation, the highest cutting speed, and the lowest feed rate. Furthermore, enlargement of printed pilot hole sizes of 9 mm and 9.5 mm in vertical orientation proved to be the most advantageous from the perspective of hole quality.

### 3.4. Exploratory Multi-Response Ranking of the Tested Conditions

A simple multi-response ranking [40,41] was performed using normalized values of |Err|, Cyl, Ra and Rz. Each response was scaled to the 0–1 range, where lower values corresponded to better quality. The exploratory composite score comprised three equally weighted quality domains: dimensional accuracy, represented by normalized |Err|; form deviation, represented by normalized cylindricity; and surface texture, represented by the mean of normalized Ra and Rz. Thus, |Err| and cylindricity were assigned weights of 1/3 each, whereas Ra and Rz were assigned weights of 1/6 each. The analysis was performed twice: first for all investigated conditions, and second for a subset limited to the machining-dominated regime. In the latter analysis, the D_start_ = 10 mm condition was excluded in order to identify the most favourable conditions for which the measured roughness was predominantly generated by machining, rather than being influenced by residual printing-induced surface texture.

**Figure 13:**
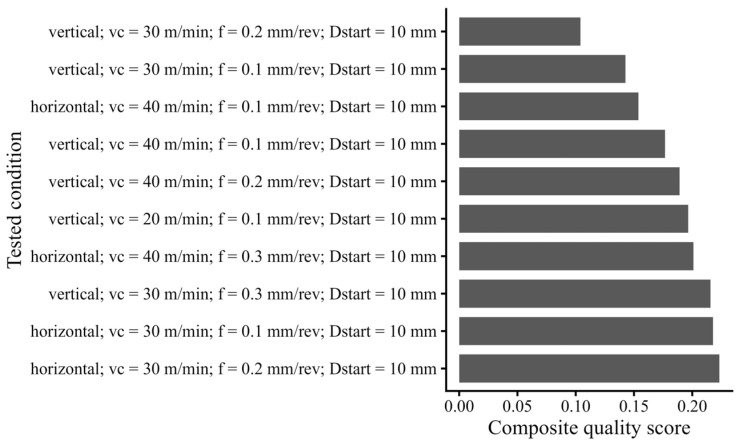
Ten tested conditions with the lowest exploratory composite quality scores (all investigated conditions).

The results of the multi-response ranking for all investigated conditions are presented in Figure 13. All ten conditions with the lowest composite quality scores were associated with the enlargement of printed pilot holes with D_start_ = 10 mm (machining allowance = 0.062 mm per side). As shown by the preceding analyses, these conditions were associated with the lowest ∣Err∣ values and the lowest cylindricity deviations. Most of these conditions were associated with the vertical build orientation. The condition with the lowest composite quality score was v_c_ = 30 m/min and f = 0.2 mm/rev.

**Figure 14:**
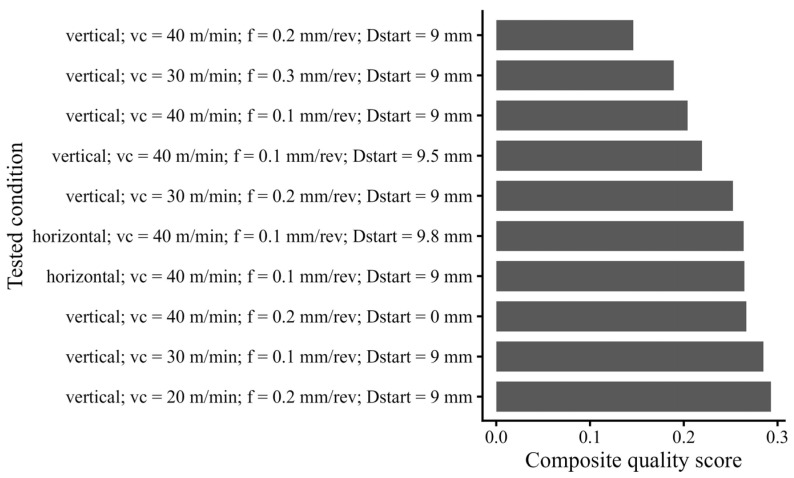
Ten tested conditions with the lowest exploratory composite quality scores for conditions limited to the machining-dominated regime.

The results of multi-response ranking for conditions limited to the machining-dominated regime are presented in Figure 14. Most of these conditions were associated with the vertical build orientation, an initial hole diameter of D_start_ = 9 mm (corresponding to a machining allowance of 0.565 mm per side), and the highest investigated cutting speed. In the present study, a larger machining allowance was only available for drilling from solid material (D_start_ = 0 mm). These results suggest that future studies should investigate machining allowances greater than 0.565 mm per side and extend the range of cutting speeds towards higher values. The lowest overall quality score, corresponding to the best overall hole quality, was obtained for specimens printed in the vertical orientation and enlargement of printed pilot holes from D_start_ = 9 mm (machining allowance of 0.565 mm per side) using a cutting speed of 40 m/min and a feed rate of 0.2 mm/rev.

When comparing the lowest-scoring conditions (v_c_ = 40 m/min, f = 0.2 mm/rev) with previously published results on the machining of MEX-manufactured components, differences can be observed that may be related to the specific characteristics of printed pilot-hole enlargement. While studies [42] on PLA parts and [14] on PC/ABS components focused on conventional drilling from solid material, they indicated that lower feed rates may be preferable to reduce delamination and structural damage around the hole edges. In contrast, the present results indicate that, for printed pilot-hole enlargement, particularly at D_start_ = 9 mm, a feed rate of 0.2 mm/rev was associated with a lower composite quality score within the tested dataset. This difference may be related to the reduced machining allowance compared with drilling from solid material, potentially limiting frictional heating and thermally induced surface smearing. Similar thermal effects during the post-processing of MEX-manufactured components have been reported in [43].

## 4. Discussion

The obtained results indicate that the mechanism of geometric error and roughness formation during the enlargement of printed pilot holes in ABS components is strongly determined by a complex interaction between the cutting conditions and the specific, layered structure of the additively manufactured material [24,43]. This process cannot be described and optimized solely in terms of classical cutting theory for homogenous metals [14,44]. Instead, it may be conceptualized as a multidimensional phenomenon involving the superposition of shearing, significant elasto-plastic deformation, and tribological phenomena occurring at the tool–material interface [27,42]. Pronounced differences in the obtained results depending on the print orientation and the initial hole diameter value (D_start_) strongly suggest that the observed trends are consistent with a possible relation between the layered material structure and stock removal [15,26].

### 4.1. Analysis of Dimensional Deviations

The analysis of linear dimensional errors (Err) consistently reveals negative values, indicating a systematic reduction in the diameter of the machined holes relative to the nominal size of the cutting tool. This can be primarily attributed to the proposed mechanism of elasto-plastic deformations and material relaxation after the passage of the cutting edge [23,45]. As a thermoplastic polymer, ABS is characterized by a relatively low stiffness, and its mechanical properties are highly dependent on temperature and loading history [25,26]. During machining, localized plasticization of the material is assumed to occur around the tool, leading to its temporary deformation followed by partial elastic relaxation. This phenomenon likely results in a specific ‘closing’ or contraction of the material behind the cutting zone [42]. Across the investigated range, increasing cutting speed shifted the predicted diameter deviation toward zero. Because the temperature in the cutting zone was not measured, the mechanism underlying this trend cannot be determined directly from the present data. Although flood cooling was applied during machining, local temperature increase in the cutting zone cannot be excluded. Local thermal softening of ABS is considered a plausible factor that may promote smearing of the polymer on the machined surface, particularly at unfavorable combinations of feed rate and machining allowance. However, since no direct thermographic measurements or cutting force analyses were performed in this study, this interpretation should be regarded as a plausible mechanism rather than a directly verified one.

Interestingly, in the case of enlargement of printed pilot holes with an initial diameter of D_start_ = 10 mm (where the material allowance is nearly zero), a completely distinct behavior is observed. The extremely thin material layer appears to cause the process to shift from a free-cutting mechanism to a regime hypothesized to be dominated by friction and the so-called ‘smearing’ effect (material rubbing), in which the share of surface contact likely increases at the expense of effective chip formation [27,46].

### 4.2. Analysis of Cylindricity Deviation

Although feed rate and cutting speed may influence thermal and mechanical loading during polymer drilling [14,20], neither factor was retained in the final cylindricity model in the present study. The primary factors explaining the cylindricity deviation are the initial diameter of the printed pilot hole (D_start_), the build orientation, and the interaction between these two parameters. The build orientation influences the outcomes due to the structural anisotropy of the MEX parts, which results from weaker interlayer bonds [47,48]. This structural characteristic alters the load transfer mechanism during the enlargement of the printed holes. Specifically, varying the tool axis relative to the layer arrangement leads to different susceptibilities to deformation and potential differences in microcrack propagation or delamination under cutting forces. This provides one possible interpretation of the D_start_ × build-orientation interaction retained in the fitted model.

The relatively low marginal and conditional coefficients of determination obtained for the cylindricity model (R^2^ = 0.44) suggest that cylindricity deviation is influenced by additional sources of variability that were not explicitly included in the regression model. Besides machining allowance and build orientation, the final cylindricity deviation may also depend on local heterogeneity of the MEX structure, interlayer bonding, residual stresses generated during 3D printing, local stiffness variations, tool deflection, and small differences in the tool–workpiece interaction. Similar observations have been reported in previous studies [15,17,25] which indicate that geometric accuracy of additively manufactured holes is governed by multiple interacting factors rather than by a limited set of process variables alone.

### 4.3. Surface Roughness Parameters Analysis

The evaluation of surface roughness (Ra and Rz parameters) following the machining process perfectly complements the macrogeometric analysis, highlighting how the depth of cut penetrates the rough structure of the 3D print [33]. It was found that machining parts with an insufficient material allowance (D_start_ = 10 mm) is inadequate to remove initial print defects, such as the regular scallops formed on the edges of the deposited polymer layers, resulting in drastically higher and highly variable roughness [18]. For the smallest machining allowance, the drill removes only a thin outer layer of material. Consequently, the original stair-stepping texture produced during the MEX process is not completely eliminated. The remaining printed surface contributes directly to the measured roughness parameters, explaining why this condition exhibits higher Ra and Rz values despite limited material removal.

Increasing the depth of cut (enlargement of printed pilot holes starting from smaller pilot diameters, e.g., 9 mm) is thought to allow the tool to penetrate into the more homogeneous structure within the core of the part, consequently improving the surface finish [14]. Furthermore, higher cutting speeds, combined with an adequately large material allowance, had a beneficial effect on surface smoothness [31,32]. Higher cutting speeds generally improved surface finish, which points to smoother chip formation and reduced mechanical damage of the deposited paths as a highly probable explanation. However, excessively high cutting speeds might also increase heat generation in polymers because of their low thermal conductivity.

Regarding the feed rate, higher values increase the uncut chip thickness, which is expected to increase cutting forces and potentially deteriorate surface finish. Conversely, excessively low feed rates may increase rubbing instead of efficient chip formation. The optimum feed therefore represents a compromise between stable chip formation and limited mechanical loading of the layered structure.

For most machining combinations, the vertical 3D print orientation yielded more desirable roughness results because the cutting edge moved parallel to the layer planes, reducing the risk of generating tear-out-like surface features at the interfaces between individual extrusion paths [15,49]. The generally lower surface roughness observed for vertically manufactured specimens may be associated with the orientation of the deposited paths relative to the cutting direction. During drilling, the cutting edge presumably interacts with a more continuous material structure, whereas in horizontally manufactured specimens it repeatedly crosses interlayer interfaces. These interfaces may locally reduce material support and potentially promote irregular chip formation, thus likely leading to higher surface roughness. Similar effects of build orientation on surface quality and mechanical responses were highlighted [15].

### 4.4. Microstructural Considerations and Material Heterogeneity

No internal microstructural characterization was performed in the present study. Therefore, the following interpretation is based on previously reported characteristics of MEX-manufactured ABS and should be regarded as a possible explanation rather than a directly verified mechanism. The machining response of MEX-manufactured ABS should be interpreted in relation to its layered and locally heterogeneous structure [26,48]. Even when a solid infill strategy and zero raster air gap are used, the material cannot be considered fully equivalent to a conventionally manufactured homogeneous polymer [14,48]. Owing to the layered structure of MEX-manufactured ABS, the cutting edge does not interact with a homogeneous material but with alternating deposited paths and inter-path interfaces. The local variation in bonding quality and material continuity is expected to influence chip formation, elastic recovery after cutting, and the final surface topography.

The internal structure may potentially contain local voids between adjacent deposited paths, interfaces between successive layers, and variations in inter-path bonding quality [25,26]. These features are assumed to affect local stiffness, heat dissipation, and the way in which the cutting edge interacts with the material [25,48]. In particular, weaker interlayer or inter-path bonding might promote local material pull-out or discontinuous removal, whereas better bonded regions could behave more similarly to bulk ABS [24,43]. This anisotropic behavior and its dependence on interlayer bonding in ABS-M30 structures is consistent with the trends characterized in publication [25].

Therefore, porosity, void content, and interlayer bonding are hypothesized to contribute to the variability observed in dimensional accuracy, cylindricity deviation, and surface roughness. Direct microstructural characterization of the machined region should be included in future studies.

### 4.5. Future Research Directions

Given the complexity and multifaceted nature of hybrid manufacturing processes combining MEX and machining, future research should focus on several key areas. There is a clear need for integrating thermographic measurements directly into the cutting zone in conjunction with dynamometry, which would enable a precise correlation between temperature gradients and cutting forces with the degree of elastic relaxation and roughness-related defects. A highly promising direction involves investigating the impact of innovative cooling methods (e.g., cryogenic cooling or minimum quantity lubrication—MQL), which could ‘freeze’ the elastic state of the polymer and minimize material smearing. Furthermore, future studies should extend this analysis to advanced 3D-printed composites (e.g., carbon fiber-reinforced ABS), where the presence of hard reinforcing phases will radically alter both the tribological conditions and the wear mechanisms of the cutting tool itself.

## 5. Conclusions

This study is the first to systematically investigate the combined effects of additive manufacturing conditions and machining parameters on the dimensional accuracy, geometric quality, and surface roughness of holes machined in ABS-M30 specimens produced using MEX technology. The main objective was to determine the influence of the initial hole diameter, build orientation, cutting speed, and feed rate on the final hole quality in a hybrid manufacturing process combining additive and subtractive technologies. The experimental investigations and statistical analyses led to the following conclusions:

- All measured diameter deviation values (Err) were negative, indicating a systematic reduction in the diameter of the machined holes relative to the nominal dimension. This behavior is consistent with the elastic–plastic deformation and spring-back effect of the ABS material during machining.
- Specimens with an initial hole diameter of D_start_ = 10 mm exhibited different behavior compared to the other variants. They were characterized by the smallest dimensional deviations and cylindricity deviation, but at the same time by the highest and most variable surface roughness values. This behavior was associated with the very small machining allowance, which was insufficient to effectively remove the surface irregularities generated during the printing process.
- Surface quality was strongly dependent on the interaction between build orientation, machining allowance, and machining parameters. Higher cutting speeds combined with lower feed rates generally produced lower Ra and Rz values. Overall, specimens printed in the vertical orientation exhibited better surface quality, although the magnitude of this effect depended on the initial hole diameter.
- Hole cylindricity deviation was primarily affected by the interaction between build orientation and pilot-hole diameter, confirming that the anisotropic structure of MEX-manufactured ABS significantly influences the machining process.
- The exploratory multi-response ranking, based on dimensional deviation, cylindricity deviation, Ra, and Rz, showed that all ten conditions with the lowest composite quality scores for the complete dataset were associated with D_start_ = 10 mm (machining allowance of 0.062 mm per side), with most of these conditions also associated with the vertical build orientation. These results reflect the favourable dimensional accuracy and cylindricity deviation observed for this low-machining-allowance condition, although the measured surface texture was also influenced by residual 3D printing-induced topography.
- Within the machining-dominated regime, several of the lowest-scoring conditions were associated with an initial hole diameter of D_start_ = 9 mm (machining allowance of 0.565 mm per side) and the highest investigated cutting speed of 40 m/min.

In summary, the quality of holes produced in MEX-manufactured ABS components depends on the interaction between machining parameters and the anisotropic material structure resulting from the 3D printing process. Therefore, the selection of hybrid manufacturing conditions should not be based solely on conventional assumptions developed for homogeneous materials but must also account for the specific characteristics of the layered structure of additively manufactured materials.

## Figures and Tables

**Table 1:** Variables analyzed in the study.

Input Variables	Value Levels/Parameter Levels
Nominal diameter of the printed pilot hole	No printed pilot hole (drilling in solid material) and 9.0, 9.5, 9.8, and 10.0 mm
Hole axis orientation during printing	Vertical, horizontal
Cutting speed	20, 30, 40 m/min
Feed rate	0.1, 0.2, 0.3 mm/rev

**Table 2:** Technological parameters of the 3D printing process for ABS-M30 samples.

Process Parameters	Value Levels/Parameter Levels
Nozzle designation (model/support)	T12/T12
Layer thickness	0.1778 mm (0.0070 in)
Interior fill style	Solid
Visible surface finish	Enhanced
Nominal contour width	0.3556 mm (0.0140 in)
Nominal raster width	0.3556 mm (0.0140 in)
Raster air gap	0.0000 mm
Contour-to-raster air gap	0.0000 mm
Support style	Sparse
Support separation interface	Break-away
Raster deposition angle	Alternating +45°/−45° in successive layers

**Table 3:** Cutting parameters used in the experimental tests.

Run No.	v_c_ [m/min]	f [mm/rev]
1	20	0.1
2	20	0.2
3	20	0.3
4	30	0.1
5	30	0.2
6	30	0.3
7	40	0.1
8	40	0.2
9	40	0.3

**Table 4:** Hole diameters in the prepared samples and calculated mean allowance.

			Nominal			
		0 mm	9.0 mm	9.5 mm	9.8 mm	10.0 mm
horizontal orientation	Mean	-	8.955	9.444	9.750	9.950
	Std	-	0.010	0.011	0.017	0.011
vertical orientation	Mean	-	8.945	9.439	9.755	9.964
	Std	-	0.024	0.017	0.016	0.033
Allowance per side		5.040	0.565	0.319	0.164	0.062

**Table 5:** Descriptive statistics for the diameter deviation (Err).

D_start_, mm	Err_Mean, mm	Err_Median, mm	Err_sd, mm
0	−0.082	−0.083	0.007
9	−0.076	−0.076	0.005
9.5	−0.074	−0.074	0.003
9.8	−0.08	−0.079	0.007
10	−0.054	−0.055	0.005

**Table 6:** Regression analysis results for the model Err = f(v_c_, f, D_start_, orientation).

Term	Estimate	Standardized	Std. Error	t Value	*p*_Value
(Intercept)	−0.0901	NA	0.0032	−28.1380	<0.0001
Orientation [vertical]	0.0071	1.1010	0.0019	3.7360	0.0004
v_c_	0.0003	0.3180	0.0001	3.1180	0.0059
f	−0.0171	−0.2170	0.0081	−2.1280	0.0474
D_start_ [9]	0.0070	1.0830	0.0016	4.3860	0.0001
D_start_ [9.5]	0.0123	1.8930	0.0016	7.6680	<0.0001
D_start_ [9.8]	0.0099	1.5240	0.0016	6.1760	<0.0001
D_start_ [9]: orientation [vertical]	−0.0018	−0.2710	0.0023	−0.7760	0.4410
D_start_ [9.5]: orientation [vertical]	−0.0073	−1.1330	0.0023	−3.2470	0.0020
D_start_ [9.8]: orientation [vertical]	−0.0143	−2.2010	0.0023	−6.3060	<0.0001

**Table 7:** Descriptive statistics for the cylindricity deviation (Cyl).

D_start_, mm	Cyl_Mean, mm	Cyl_Median mm	Cyl_sd, mm
0	0.021	0.021	0.005
9	0.017	0.016	0.005
9.5	0.026	0.025	0.004
9.8	0.021	0.02	0.004
10	0.006	0.005	0.002

**Table 8:** Model Cyl = f(D_start_, orientation).

Term	Estimate	Standardized	Std. Error	t Value	*p*_Value
(Intercept)	0.0184	NA	0.0014	13.3730	<0.0001
D_start_ [9]	0.0014	0.2560	0.0019	0.7280	0.4688
D_start_ [9.5]	0.0071	1.2840	0.0019	3.6470	0.0005
D_start_ [9.8]	0.0034	0.6080	0.0019	1.7280	0.0883
Orientation [vertical]	0.0053	0.9560	0.0019	2.7220	0.0081
D_start_ [9]: orientation [vertical]	−0.0106	−1.9120	0.0028	−3.8390	0.0003
D_start_ [9.5]: orientation [vertical]	−0.0046	−0.8290	0.0028	−1.6640	0.1004
D_start_ [9.8]: orientation [vertical]	−0.0066	−1.1990	0.0028	−2.4070	0.0186

**Table 9:** Descriptive statistics for the Ra parameter.

D_start_, mm	Ra_Mean, µm	Ra_Median, µm	Ra_sd, µm
0	1.844	1.677	0.652
9	1.597	1.562	0.614
9.5	2.121	2.028	1.112
9.8	2.276	2.046	1.064
10	3.058	2.9	1.575

**Table 10:** Descriptive statistics for the Rz parameter.

D_start_, mm	Rz_Mean, µm	Rz_Median, µm	Rz_sd, µm
0	12.453	12.276	4.506
9	10.831	9.033	4.313
9.5	14.046	12.125	6.756
9.8	15.493	14.725	6.109
10	20.697	20.572	8.113

**Table 11:** Model log(Ra) = f(v_c_, f, D_start_, orientation).

Term	Estimate	Standardized	Std. Error	t Value	*p*_Value
(Intercept)	0.3559	NA	0.2123	1.6760	0.0980
v_c_	−0.0127	−0.2150	0.0050	−2.5560	0.0127
f	2.5373	0.4310	0.4958	5.1180	<0.0001
D_start_ [9]	0.0874	0.1810	0.1619	0.5400	0.5910
D_start_ [9.5]	0.4839	1.0000	0.1619	2.9880	0.0038
D_start_ [9.8]	0.3243	0.6700	0.1619	2.0030	0.0489
Orientation [vertical]	0.1471	0.3040	0.1619	0.9080	0.3668
D_start_ [9]: orientation [vertical]	−0.5013	−1.0350	0.2290	−2.1890	0.0318
D_start_ [9.5]: orientation [vertical]	−0.8965	−1.8520	0.2290	−3.9150	0.0002
D_start_ [9.8]: orientation [vertical]	−0.3441	−0.7110	0.2290	−1.5030	0.1373

**Table 12:** Model log(Rz) = f(v_c_, f, D_start_, orientation).

Term	Estimate	Standardized	Std. Error	t Value	*p*_Value
(Intercept)	2.4418	NA	0.2072	11.7860	<0.0001
v_c_	−0.0137	−0.2440	0.0051	−2.7020	0.0146
f	2.3372	0.4160	0.5069	4.6110	0.0002
D_start_ [9]	0.0618	0.1340	0.1275	0.4850	0.6298
D_start_ [9.5]	0.3961	0.8590	0.1275	3.1070	0.0030
D_start_ [9.8]	0.2817	0.6110	0.1275	2.2090	0.0314
Orientation [vertical]	−0.0662	−0.1430	0.1380	−0.4800	0.6328
D_start_ [9]: orientation [vertical]	−0.4503	−0.9760	0.1803	−2.4970	0.0156
D_start_ [9.5]: orientation [vertical]	−0.7173	−1.5550	0.1803	−3.9780	0.0002
D_start_ [9.8]: orientation [vertical]	−0.1747	−0.3800	0.1803	−0.9690	0.3368

## Data Availability

The original contributions presented in this study are included in the article. Further inquiries can be directed to the corresponding author.

## References

[1] Lacroix R., Seifert R.W., Timonina-Farkas A. (2021). Benefiting from additive manufacturing for mass customization across the product life cycle. Oper. Res. Perspect..

[2] Patel A., Taufik M. (2024). Extrusion-based technology in additive manufacturing: A comprehensive review. Arab. J. Sci. Eng..

[3] Jayashuriya M., Gautam S., Aravinth A.N., Vasanth G., Murugan R. (2022). Studies on the effect of part geometry and process parameter on the dimensional deviation of additive manufactured part using ABS material. Prog. Addit. Manuf..

[4] Mittal Y.G., Patil Y., Kamble P.P., Gote G.D., Mehta A.K., Karunakaran K.P. (2024). Warpage control in thermoplastic ABS parts produced through material extrusion (MEX)-based fused deposition modeling (FDM). Rapid Prototyp. J..

[5] Musa N.H., Mohd Yusuf S., Abu Mansor M.R., Abdul Kadir A.Z., Suhaimi M.A., Gao N., Yang S. (2025). Material extrusion (MEX)-based additive manufacturing of metallic materials: Review of current trends, challenges, and opportunities. Int. J. Adv. Manuf. Technol..

[6] Machiels J., Verma A., Appeltans R., Buntinx M., Ferraris E. (2023). Printed Electronics (PE) as an enabling technology to realize flexible mass customized smart applications. Procedia CIRP.

[7] Ngo T.D., Kashani A., Imbalzano G., Nguyen K.T.Q., Hui D. (2018). Additive manufacturing (3D printing): A review of materials, methods, applications and challenges. Compos. Part B Eng..

[8] Ravi P., Chepelev L.L., Stichweh G.V., Jones B.S., Rybicki F.J. (2022). Medical 3D printing dimensional accuracy for multi-pathological anatomical models 3D printed using material extrusion. J. Digit. Imaging.

[9] Mora S.M., Gil J.C., López A.M.C. (2019). Influence of manufacturing parameters in the dimensional characteristics of ABS parts obtained by FDM using reverse engineering techniques. Procedia Manuf..

[10] Hernandez D.D. (2015). Factors affecting dimensional precision of consumer 3D printing. Int. J. Aviat. Aeronaut. Aerosp..

[11] Vyavahare S., Kumar S., Panghal D. (2020). Experimental study of surface roughness, dimensional accuracy and time of fabrication of parts produced by fused deposition modelling. Rapid Prototyp. J..

[12] Park S.J., Lee J.S., Lee J.E., Moon S.K., Son Y., Park S.H. (2024). Influence of nozzle temperature on gas emissions and mechanical properties in material extrusion-based additive manufacturing of super engineering plastics. Int. J. Precis. Eng. Manuf. Green Technol..

[13] Gonzalez-Gutierrez J., Cano S., Ecker J.V., Kitzmantel M., Arbeiter F., Kukla C., Holzer C. (2021). Bending properties of lightweight copper specimens with different infill patterns produced by material extrusion additive manufacturing, solvent debinding and sintering. Appl. Sci..

[14] Mavi F., Ayan K., Aslan N., Polat S.C., Saklakoğlu İ.E., Saklakoğlu N. (2025). Drilling optimisation for additively manufactured PC/ABS parts: Effects of shell layer count in the MEX process. J. Adv. Manuf. Eng..

[15] Greco A., Russo M.B., Gerbino S. (2025). Simultaneous impact of build orientation on mechanical properties, geometrical measurements and surface roughness in material extrusion manufacturing. Rapid Prototyp. J..

[16] Akbaş O.E., Hıra O., Hervan S.Z., Samankan S., Altınkaynak A. (2020). Dimensional accuracy of FDM-printed polymer parts. Rapid Prototyp. J..

[17] Popescu D., Amza G., Marinescu R., Iacob M.C. (2023). Investigations on Factors Affecting 3D-Printed Holes Dimensional Accuracy and Repeatability. Appl. Sci..

[18] Rebaioli L., Fassi I. (2017). Performance of additive manufacturing processes. Int. J. Adv. Manuf. Technol..

[19] Bakardzhiev V., Sabev S., Chukalov K., Kasabov P. (2023). Research into the Accuracy of Holes in 3D Printing Using Taguchi Method. Environ. Technol. Resour..

[20] Kumar A.M., Jayakumar K. (2022). Mechanical and drilling characterization of biodegradable PLA particulate green composites. J. Chin. Inst. Eng..

[21] Baraheni M., Shabgard M.R. (2024). Experimental evaluation and optimization of parameters affecting delamination, geometrical tolerance and surface roughness in ultrasonic drilling of 3D-Printed PLA thermoplastic. J. Thermoplast. Compos. Mater..

[22] Borysiuk P., Auriga R., Wilkowski J., Auriga A., Trociński A., Seng Hua L. (2022). A Study on the Susceptibility of PLA Biocomposites to Drilling. Forests.

[23] Santus C., Neri P., Romoli L. (2024). Residual Stress Determination with the Hole-Drilling Method on FDM 3D-Printed Precurved Specimen through Digital Image Correlation. Appl. Sci..

[24] Khosravani M.R., Schüürmann J., Berto F., Reinicke T. (2021). On the Post-Processing of 3D-Printed ABS Parts. Polymers.

[25] Croccolo D., De Agostinis M., Olmi G. (2013). Experimental characterization and analytical modelling of the mechanical behaviour of fused deposition processed parts made of ABS-M30. Comput. Mater. Sci..

[26] Ziemian C.W., Crawn M.M., Ziemian S.R. (2012). Anisotropic mechanical properties of ABS parts fabricated by fused deposition modeling. Mech. Eng..

[27] Carta M., Loi G., El Mehtedi M., Buonadonna P., Aymerich F. (2025). Improving surface roughness of FDM-printed parts through CNC machining: A brief review. J. Compos. Sci..

[28] Juneja S., Chohan J.S., Kumar R., Sharma S., Ilyas R.A., Asyraf M.R.M., Razman M.R. (2022). Impact of process variables of acetone vapor jet drilling on surface roughness and circularity of 3D-printed ABS parts: Fabrication and studies on thermal, morphological, and chemical characterizations. Polymers.

[29] Vora H.D., Sanyal S. (2020). A comprehensive review: Metrology in additive manufacturing and 3D printing technology. Prog. Addit. Manuf..

[30] Roithmeier R. (2014). Measuring Strategies in Tactile Coordinate Metrology.

[31] (2011). Geometrical Product Specifications (GPS)—Cylindricity—Part 1: Vocabulary and Parameters of Cylindrical Form.

[32] (2011). Geometrical Product Specifications (GPS)—Cylindricity—Part 2: Specification Operators.

[33] (2021). Geometrical Product Specifications (GPS)—Surface Texture: Profile—Part 2: Terms, Definitions and Surface Texture Parameters.

[34] Leach R. (2013). Characterisation of Areal Surface Texture.

[35] Ouazzani K., El Jai M., Akhrif I., Radouani M., El Fahime B. (2023). An experimental study of FDM parameter effects on ABS surface quality: Roughness analysis. Int. J. Adv. Manuf. Technol..

[36] R Core Team (2024). R: A Language and Environment for Statistical Computing.

[37] Van Rossum G., Drake F.L. (2009). Python 3 Reference Manual.

[38] Hunter J.D. (2007). Matplotlib: A 2D Graphics Environment. Comput. Sci. Eng..

[39] Waskom M.L. (2021). Seaborn: Statistical data visualization. J. Open Source Softw..

[40] Burnham K.P., Anderson D.R. (2001). Model Selection and Multimodel Inference: A Practical Information-Theoretic Approach.

[41] Konishi S., Kitagawa G. (2010). Information Criteria and Statistical Modeling.

[42] Köklü U., Urtekin L., Akdoğan E., Yılan F., Demiral M. (2026). Experimental investigation and numerical validation of drilling machinability in FDM-printed PLA parts. J. Manuf. Process..

[43] Alzyod H., Kónya G., Ficzere P. (2025). Integrating additive and subtractive manufacturing to optimize surface quality of MEX parts. Results Eng..

[44] Giraud C. (2015). Introduction to High-Dimensional Statistics.

[45] Davim J.P. (2013). Machining and Machine-Tools: Research and Development.

[46] Sun F., Fu G., Huo D. (2024). Computational and experimental analysis of surface residual stresses in polymers via micro-milling. Polymers.

[47] Gómez-García D., Díaz-Álvarez A., Youssef G., Miguélez H., Díaz-Álvarez J. (2023). Machinability of 3D printed PEEK reinforced with short carbon fiber. Compos. Part C Open Access.

[48] Sood A.K., Ohdar R.K., Mahapatra S.S. (2010). Parametric appraisal of mechanical property of fused deposition modelling processed parts. Mater. Des..

[49] Del Sol I., Domínguez Calvo Á., Piñero D., Salguero Gómez J., Batista M. (2019). Study of the FDM parameters of the ABS parts in the surface quality after machining operations. Key Eng. Mater..

